# Barriers to adult vaccination in Canada: A qualitative systematic review

**DOI:** 10.1177/17151635221090212

**Published:** 2022-06-06

**Authors:** Doris Stratoberdha, Barbara Gobis, Adrian Ziemczonek, Jamie Yuen, Annita Giang, Peter J. Zed

**Affiliations:** Faculty of Pharmaceutical Sciences, the University of British Columbia, Vancouver, BC; Faculty of Pharmaceutical Sciences, the University of British Columbia, Vancouver, BC; Faculty of Pharmaceutical Sciences, the University of British Columbia, Vancouver, BC; Faculty of Pharmaceutical Sciences, the University of British Columbia, Vancouver, BC; Faculty of Pharmaceutical Sciences, the University of British Columbia, Vancouver, BC; Faculty of Pharmaceutical Sciences and the Faculty of Medicine, the University of British Columbia, Vancouver, BC

## Abstract

**Background:**

In recent years, Canadian health care professionals have observed an increase in vaccine refusal. The objective of this study is to review published literature and identify the main themes related to vaccine hesitancy and barriers to vaccination in Canadian adults and recent immigrants.

**Methods:**

A qualitative systematic review was performed. A comprehensive search of MEDLINE (1946 to January 2021) and EMBASE (1974 to January 2021) was conducted to identify existing literature that addressed the primary research question. Studies were eligible for inclusion if the study population involved 1) the general population, 2) Indigenous populations, 3) recent immigrants to Canada or 4) Canadian health care professionals.

**Results:**

Thirty-four studies were included with a focus on the general population (*n* = 22), health care professionals (*n* = 10) and recent immigrant populations (*n* = 2). The most frequently reported barriers were lack of vaccine information (41%), lack of access to vaccination (38%), fear of adverse reactions (38%), financial reasons (29%), lack of awareness of vaccine existence (29%), antivaccine sentiments (24%), notion that older adults do not need vaccination (18%), misconceptions on vaccine effectiveness (12%), potential sexual health promotion stigma (6%) and fear of needles (3%).

**Interpretation:**

Barriers to vaccination among Canadians and recent immigrants continue to be a challenge in the health care system.

**Conclusions:**

The greatest yield in improving vaccination rates is likely to come from supporting vaccine-hesitant individuals in shifting their thinking to greater vaccine acceptance. Pharmacists are well positioned to address vaccine hesitancy and involvement through education, facilitation and administration of vaccines. *Can Pharm J (Ott)* 2022;155:xx-xx.

## Introduction

Vaccines and vaccination programs prevent millions of deaths annually around the world.^1^ In Canada, vaccines have eliminated, contained and controlled diseases that were once very common.^2^ Unfortunately, vaccine hesitancy is on the rise in many countries, including Canada.^3^ The World Health Organization defines vaccine hesitancy as suboptimal vaccination coverage due to delay in acceptance or refusal of vaccines despite availability of vaccination services.^4^ A 2018 survey of Canadian health care professionals reported that 26% of family physicians have observed an increase in vaccine refusal over the past 5 years.^5^ The measles, mumps and rubella (MMR) vaccine had the highest rate of refusal, and the most common reason for refusal is knowing someone who has experienced an adverse effect from a vaccine.^5^ Infants and children are particularly susceptible to vaccine-preventable diseases, and guidance is available for medical professionals to address parental vaccine hesitancy in primary care.^6^ Immunizations are important for adults to restore waning immunity and to build new immunity against diseases that are more common in adults.^7^

During the coronavirus disease 2019 (COVID-19) pandemic, understanding and addressing vaccine hesitancy to increase individual and herd immunity are important as vaccines become increasingly available. This article reviews the published literature and identifies the main themes related to vaccine hesitancy and barriers to vaccination in Canadian adults and recent immigrants to Canada. These findings can inform future vaccination initiatives and messages for undervaccinated Canadian adults.

## Methods

This qualitative systematic review was performed and reported in compliance with the recommendations from the Preferred Reporting of Systematic Reviews and Meta-Analyses (PRISMA).^8^

### Search strategy

A comprehensive search of MEDLINE (1946 to January 2021) and EMBASE (1974 to January 2021) was conducted to identify existing literature that addressed the primary research question. The search terms and Boolean used in MEDLINE and EMBASE are outlined in Appendix 1.

### Selection of studies

Studies of vaccine hesitancy and barriers to vaccination in Canada were eligible for inclusion if the study population involved 1) the general population, 2) Indigenous populations, 3) recent immigrants to Canada and 4) Canadian health care professionals. Exclusion criteria applied during preliminary screening were studies involving 1) pediatric patients, 2) parents’ vaccine hesitancy and barriers towards vaccinating their children, 3) specific conditions (e.g., pregnancy, breastfeeding, rheumatoid arthritis, asthma) and 4) specific populations (e.g., sex workers). Two authors (DS, BG) screened the search results for potentially relevant studies. Potentially relevant citations were then reviewed in full to determine whether the predefined inclusion criteria were met.

### Data extraction and management

Data from each included study were extracted and tabulated. Extracted data included authors, language, study design, year of publication, type of vaccine, study population, barriers to vaccination and limitations. Data were first extracted by 1 author (DS), then checked by the second (BG); disagreements or inconsistencies regarding data extraction were resolved by discussion and consensus.

### Data analysis

Descriptive analyses were performed on data extracted from the included studies.

## Results

The search results and flow diagram are outlined in Appendix 2, available at www.cpjournal.ca. The initial search yielded 428 studies, of which a total of 34 studies met the criteria and were included in this review (Table 1).^9-42^ Among included studies, 22 (65%) focused on the general population, 10 (29%) focused on health care professionals and 2 (6%) focused on recent immigrant populations.

**Table 1:** Characteristics of included studies

Study and year	Research question	Study design	Context/setting/sample	Barriers identified	Limitations
McIntyre et al., 2014^9^	Self-perceived influences among older adults in deciding whether to take or not take the seasonal influenza vaccine	Six 60-minute focus group interviews	Southwestern Ontario *N* = 37 31 receivers and 6 nonreceivers of influenza vaccine Age >65, average age 82 years	Fear of adverse reactions Perceptions of resilience to the vaccine-preventable disease	Free access to influenza vaccinations in study location Vaccine uptake is greater in retirement homes Limited generalizability
Boerner et al., 2013^11^	Vaccinating behaviours, the impact of public health messaging and the public’s attitudes toward H1N1 and the H1N1 vaccine	Fifteen focus group interviews	Ontario, Alberta and Manitoba *N* = 143 Equal size groups with ages: 18–34, 35–55 and 55+ years	Access to vaccination	Recall bias Potential for statements to be influenced by focus group members and discussion
Taddio et al., 2012^14^	Prevalence of needle fear in adults and children and the impact of needle fear on vaccine compliance	Cross-sectional survey	Ontario *N* = 2007; 883 parents & 1024 children Inclusion criteria: English speaking and experience with immunization	Fear of needles	Reporting bias Study sample average education level higher than regional average Possible underreporting
Roy et al., 2018^17^	Identify health and socio-demographic factors associated with nonvaccination	Canadian community health survey	Canada *N* = 108,700 divided into 3 groups: Adults aged 18 to 64 years with a chronic medical condition Adults aged ≥65 years Adults aged 18 to 64 years with no chronic medical condition	Perceptions of resilience to the vaccine-preventable disease Antivaccine sentiments	Recall bias Seniors in long-term care facilities and people living on Aboriginal reserves excluded Vaccination status based off 1 flu season
Kiberd et al., 2010^22^	Explore attitudes and behaviours of Canadian adults regarding recommended vaccines	Web-based Canada-wide survey	Canada *N* = 4067 47.5% were 18–44, 35% 45–64 & 17.5% ≥65 years	Access to vaccination Vaccine awareness Lack of vaccine information	Limited to individuals with Internet access Recall bias
Rousseau et al., 2007^24^	Explore the presence of barriers in relation to the organization of the health care system and to propose recommendations for increasing vaccine coverage	Telephone survey and small discussion groups	Quebec *N* = 996 Health care workers 22%, parents of children with chronic illness 11%, adults aged <60 with chronic disease 36%, people aged ≥60 31%	Access to vaccination Lack of vaccine information	Recall bias Selection bias
Ozog et al., 2019^27^	Gauge public interest, HCP support, perceived barriers and perceived facilitators to influenza vaccine availability at ED	Short, anonymous, close-ended questionnaires over a 7-week period	Nova Scotia *N* = 230 150 adult clients who use the ED during the study period and 80 health care professionals currently working at the ED	Access to vaccination	Study and results limited to ED setting
Halperin et al., 2015^39^	Explore the knowledge, attitudes, beliefs and behaviours of the Canadian public regarding pertussis and pertussis vaccination	Web-based nationwide survey, a self-administered questionnaire format	Canada *N* = 4023 Subset of adults based on regional representation across the country, age, gender and urban and rural residence Participants were ≥18 years and had Internet access	Vaccine awareness Lack of vaccine information	Recall bias
MacDougall et al., 2015^40^	Explore the knowledge, attitudes and behaviours of Canadian HCPs to identify barriers and facilitators to Tdap uptake	Survey, 8 focus groups and 4 interviews	Canada *N* = 1167 Family physicians 42.8%, internists 5.6%, pharmacists 34.3%, nurses 17.3% Focus groups *N* = 45 Family physicians 36%, pharmacists 27%, nurses 24%, general internists 4%, pediatricians 4%	Antivaccine sentiment Access to vaccination Financial reasons Lack of vaccine information	Potential for statements to be influenced by focus group members and discussion
Prematunge et al., 2014^42^	Identify key motivators and barriers of HCWs to influenza vaccination in pandemic influenza and seasonal influenza settings	Survey packages	Ontario *N* = 3275 Nurses 35.2%, physicians 5.3%, allied HCWs 11.0%, administrative/clerical 22.0%, health care technicians 7.4%, research and laboratory 8.4%, facilities and logistics 6.6%, other nonclinical 4.1%	Fear of adverse reaction Lack of vaccine information	Sampling bias Overrepresentation of vaccinated HCWs Reported vaccination motivators and barriers may be somewhat subjective Lacks sensitivity to subtle barriers and motivators guiding HCW vaccination
Zibrik et al., 2018^33^	Identify the impact of culturally relevant information and challenges with recommendations for effective public education and outreach programs	Pre- and postworkshop surveys and interviews	British Columbia *N* = 827 HBV education workshop participants (Chinese, Filipino, Korean and Punjabi immigrants)	Access to vaccination Vaccine awareness Lack of vaccine information	Limited generalizability
Corace et al., 2013^12^	Identify the motivators and barriers to pH1N1 vaccine uptake among HCWs	Cross-sectional survey	Ontario *N* = 3275 Female 81%, white 89%	Fear of adverse reaction Perceptions of resilience to the vaccine-preventable disease Effectiveness misconceptions Vaccine awareness	Recall bias Limited generalizability Sampling bias
Quach et al., 2013^10^	Strategies to achieve high immunization coverage in HCWs, barriers to uptake and perceptions of mandatory influenza immunization policies	Telephone interviews	Canada *N* = 44 23 influenza immunization program planners from 21 organizations	Fear of adverse reactionEffectiveness misconceptions Negative personal experiences Antivaccine sentiments	Themes explored were not preidentified and emerged during data analysis after interviews had been completed. Not all provinces and territories represented Limited generalizability
Perez et al., 2013^13^	Examine knowledge, attitudes and beliefs about HPV and the HPV vaccine among a sample of Canadian males	Anonymous online questionnaire	Quebec *N* = 61 males with mean age of 20.7 years	Vaccine awareness	Small sample size Limited generalizability
Pullagura et al., 2020^15^	Understand community pharmacists’ attitudes towards and experiences with influenza VH and explore factors impacting their engagement with patients on the influenza vaccine	Semistructured interviews	Ontario *N* = 22 Most authorized to administer injections (*n* = 20, 90.9%) and practised for >20 years (*n* = 16, 72.7%)	Antivaccine sentiment Access to vaccination	Results reflect subjective experiences No formal assessment of interrater reliability Researcher bias Social desirability bias
Piedimonte et al., 2018^16^	Determine the level of knowledge related to HPV and cervical cancer among university students and to subsequently develop a targeted education and vaccination campaign to increase uptake	Self-administered questionnaire	Quebec *N* = 56 Participants responded to a questionnaire; among these, 29 were vaccinated in a 2-day resident-run clinic	Access to vaccination Financial reasons Perceptions of resilience to the vaccine-preventable disease	Low participation and ability to provide continuous vaccination advertising
Corace et al., 2011^18^	Examine the motivators and barriers influencing pH1N1 vaccination among HCWs to design and implement a more effective vaccine campaign that addresses these barriers	Mail-out survey	Ontario *N* = 3260 2848 who received the pH1N1 vaccine and 412 who refused	Fear of adverse reaction Perceptions of resilience to the vaccine-preventable disease Effectiveness misconception Vaccine awareness	Limited generalizability
Giede et al., 2010^19^	Identify gaps in knowledge of the link between HPV infection, cervical dysplasia and cervical cancer among women attending the Student Health Services and to identify barriers to HPV vaccination among this cohort of women	18-question survey	Saskatchewan *N* = 400 surveys distributed and 371 (91%) were returned	Fear of adverse reaction Financial reasons Lack of vaccine information	Limited generalizability
Slaunwhite et al., 2009^20^	Increase awareness of the benefits associated with influenza vaccination	Sample *t*-test used to analyze difference between groups	Nova Scotia *N* = 23	Vaccine awareness	Limited generalizability
Corace et al., 2011^23^	Design and implement more effective vaccine campaigns	Mail-out survey	Ontario *N* = 3260 2848 who received the pH1N1 vaccine and 412 who refused	Fear of adverse reaction Perceptions of resilience to the vaccine-preventable disease Effectiveness misconception	Limited generalizability
Pielak et al., 2003^25^	Compare students who were immunized or not immunized during the 1997 measles outbreak in British Columbia	Self-administered questionnaire	British Columbia *N* = 400 immunized and 400 nonimmunized university students	Fear of adverse reaction Access to vaccination Financial reasons	Attitudes and beliefs regarding measles may have differed before, during and after the measles outbreak. Variables comprising data collection instruments were not measured to provide proper resolution of dimensionality.
Steben et al., 2019^26^	Identify HPV vaccination motivators and barriers among adults to lead to new approaches to improve HPV vaccination rates in nonpediatric populations	Online 16-item questionnaire	Canada *N* = 1252 802 HPV unvaccinated women and 250 vaccinated women aged 18 to 45 years, 200 men aged 18 to 26 years	Fear of adverse reaction Antivaccine sentiment Financial reasons Lack of vaccine information	Selection bias Unvaccinated women surveyed were on average older than the vaccinated women and responses may reflect age cohort trends and differential targeting of health information among groups
Steben et al., 2019^28^	Explore knowledge, barriers and preventive practices regarding HPV vaccination	Survey using online panel	Canada *N* = 418 337 GPs, 81 OB/GYN	Financial reasons	Response bias Survey administered before the release of July 2016 NACI guidelines with updated guidance on vaccine benefits
Fernandes et al., 2018^29^	Determine the acceptability of catch-up HPV vaccination to undergraduate university women under the age of 25 by assessing their perceptions of HPV vaccination	Cross-sectional bilingual web-based survey	Ontario *N* = 401 female undergraduate students	Fear of adverse reactionFinancial reasons Lack of vaccine information	Response rate of survey was 17% Self-reported information with potential for recall and reporting errors
McComb et al., 2018^30^	Explore reasons for lower uptake of HPV vaccine among new emigrants and refugees	Semistructured interviews	Saskatchewan *N* = 11 immigrant women, 18 to 26 years old	Perceptions of resilience to the vaccine-preventable disease Lack of vaccine information	Potential researcher bias Potential social desirability bias
Mrklas et al., 2018^31^	Appraise the literature in Canadian and global Indigenous peoples, relating to documented barriers and supports to vaccination and interventions to increase acceptability/uptake or reduce hesitancy of vaccination	Systematic review of studies	Alberta Eligible studies include global Indigenous peoples and discuss barriers or supports and/or interventions to improve uptake or to reduce hesitancy for the HPV vaccine and/or other vaccines	Financial reasons	
Henderson et al., 2018^32^	Identify ways to increase HPV vaccination among people living in FN communities	Group dialogue	Alberta *N* = 24 community elders, parents, health directors and cancer survivors	Negative personal experience Access to vaccination Potential sexual health promotion stigma	Not a population-based cohort
Tatar et al., 2017^35^	Evaluate the psychosocial correlates of HPV acceptability in college males, based on multiple stages of HPV decision-making	Online questionnaire	Quebec *N* = 428 College men aged 18 to 26	Vaccine awareness Lack of vaccine knowledge	
Scott **and Batty**, 2016^37^	Investigate factors related to HPV vaccine uptake in Canada and explore role of NPs in collaborating with public health agencies to expand knowledge and coverage of the HPV vaccine across Canada	Literature review	New Brunswick 4 electronic databases searched (PubMed, Google Scholar, Cumulative Index to Nursing and Allied Health Literature [CINAHL] and Medline)	Antivaccine sentiment Lack of vaccine information	
Jones et al., 2016^38^	Identify socio-demographic and psychosocial predictors of HPV-related stigma and examine the relationship between HPV-related stigma in predicting HPV vaccine decision-making among college males	Self-reported survey	Canada *N* = 680 College males aged 18 to 26	Vaccine awareness Potential sexual health promotion stigma	Study design does not allow the identification of causal relationships or the evaluation of changes over time Sampling methods used limit generalizability Study sample composed of younger college males compared to national average Many ethnic groups may be under-represented, including African Americans (5.3% in the current sample)
**MacDougall** et al., 2015^41^	Assess the knowledge, attitudes, beliefs and behaviours of adults and health care providers related to 4 vaccine-preventable diseases and vaccines	Survey and focus groups	Canada *N* = 4023 general public’s survey *N* = 62 general public’s focus groups *N* = 1167 providers’ survey *N* = 45 providers’ focus groups	Effectiveness misconceptions Access to vaccination Vaccine awareness	Self-reporting of vaccine coverage status
Pullagura et al., 2018^34^	Understand practising CP attitudes towards influenza VH, behaviour with those hesitant to receive the influenza vaccine and experiences with influenza VH at the community pharmacy	Telephone interviews	Ontario *N* = 22 CPs where 91% were certified to provide injections and 80% had >20 years of experience	Access to vaccination	
Steben et al., 2017^36^	A national survey of Canadians on HPV: comparing knowledge, barriers and preventive practices of physicians to those of consumers “To explore knowledge, barriers and preventive practices regarding HPV”	Survey	Canada *N* = 418 physicians (*n* = 337 GPs and *n* = 81 OB/GYNs) *N* = 1139 women 18 to 45 years old (*n* = 337 vaccinated, *n* = 802 unvaccinated) *N* = 200 men between 18 and 26 years old	Fear of adverse reaction Financial reasons Lack of vaccine knowledge	
Giede et al., 2010^21^	Identify knowledge gaps regarding the link between HPV infection, cervical dysplasia and cervical cancer, as well as barriers to HPV vaccination	21-question survey	Saskatchewan *N* = 400 Survey response rate 91% (371 responses)	Fear of adverse reaction Financial reasons Lack of vaccine information	The participants were only from the University of Saskatchewan so the results cannot be generalized to the entire Canadian population.

CP, community pharmacist; ED, emergency department; FN, First Nations; GP, general practitioner; HBV, hepatitis B virus; HCP, health care provider; HCW, health care worker; HPV, human papillomavirus; NACI, National Advisory Committee on Immunization; NP, nurse practitioner; OB/GYN, obstetrician/gynecologist; Tdap, tetanus, diphtheria and pertussis; VH, Vaccine hesitancy.

Of the 34 studies included in this review, 14 focused on barriers to human papillomavirus (HPV) vaccination and 12 on barriers to influenza vaccination. The remaining studies focused on multiple vaccines (4); tetanus, diphtheria and pertussis (Tdap) vaccine (2); hepatitis B vaccine (1) and measles vaccine (1).

The most frequently reported barriers were lack of vaccine information (41%), lack of access to vaccination (38%), fear of adverse reactions (38%), financial reasons (29%), lack of awareness of vaccine existence (29%), antivaccine sentiments (24%), the notion that older adults do not need vaccination (18%), misconceptions on vaccine effectiveness (12%), potential sexual health promotion stigma (6%) and fear of needles (3%).

## Discussion

Current estimates are that up to 5% of the Canadian adult population has strong antivaccination views, while an additional 20% to 30% can be described as vaccine hesitant.^43^ Vaccine hesitancy results in the refusal or delay in receiving vaccination.^4^ Factors that contribute to vaccine hesitancy are a person’s lack of confidence or trust in the vaccine and/or health care provider; complacency, where the person does not see a need for the vaccine or does not see the value of the vaccine; fear of needles, blood or side effects from vaccines; and perceived inconvenient access to vaccines.^44^ The greatest yield in improving vaccination rates is likely to come from supporting the significant number of vaccine-hesitant individuals in shifting their thinking to greater vaccine acceptance, rather than expending disproportionate effort on the relatively small number of people with strong antivaccination views. This review revealed that a lack of vaccine information, poor access to vaccination, fear of adverse reactions, financial barriers, poor awareness of vaccine existence and antivaccine sentiments as the most common reasons for vaccine hesitancy.

Strategies and interventions aimed to address the barriers and themes identified in this study have been well described by various groups in the past. As part of their COVID-19 Working Group, the Royal Society of Canada outlines the responsibility shared by health care providers to actively support vaccine acceptance in their communities.^45^ As accessible and trusted health care providers, pharmacists are well positioned to address vaccine hesitancy by providing patient education based on the best available evidence and discussing risks and benefits associated with vaccination.^46^ Pharmacist involvement through education, facilitation and administration of vaccines has been shown to increase patient uptake of vaccination.^47^ Prioritizing these roles and implementing strategies targeting known barriers and determinants of vaccine hesitancy could help to improve vaccine acceptance (Box 1).

Box 1 **Strategies to target known barriers and determinants of vaccine hesitancy**

• Prioritize role as vaccine educators and be prepared to provide information, correct misinformation and dispel myths.

 ○ Become familiar with the factors that contribute to vaccine hesitancy and how to best support patients depending on the specific concern or perceived barrier.

 ○ Ask open-ended questions and actively listen in order to identify specific patient concerns to ensure information is tailored appropriately.

 ○ Ensure you are current on latest vaccine information and be prepared to discuss benefits and risks of vaccines.

 ○ Present strong recommendations and share personal stories and experiences when able.

 ○ Have up-to-date and credible information readily available to provide patients.

• Be proactive in starting conversations about vaccines, making this a routine part of your practice.

 ○ Ask about vaccination status during all encounters and care activities and implement an immunization assessment to identify unmet needs.

 ○ Involve all pharmacy staff members in vaccine promotion by asking initial screening questions or identifying patients who have vaccine questions.

 ○ Adopt the mind-set that individuals are underimmunized unless you can confirm otherwise and hold the expectation that vaccination is the most probable outcome.

• Ensure vaccination services are convenient and easy to access.

 ○ Provide options for both prebooked appointments and walk-ins, or refer to a provider who can administer a vaccine in a timely manner.

 ○ Keep a small quantity of routinely requested vaccines on hand to provide vaccinations opportunistically.

 ○ Offer additional routine immunizations when providing annual influenza vaccines.

### Lack of vaccine information

The most commonly cited barrier to vaccination in Canadian adults is lack of vaccine information. Participants were aware of the existence of a vaccine but had no further information on its efficacy and safety, where to receive it, the cost or if it was necessary at all. Canadians have unrestricted access to high-quality, evidence-based vaccine information written in patient-friendly language, available from organizations such as Immunize Canada and the Public Health Agency of Canada.^48,49^ Stakeholders should be focused on engaging Canadian adults in taking an interest in and seeking information about vaccines. Using a combination of interventions, such as face-to-face communication, health care provider training, community-based actions and mass media messages, appears to be much more effective than single-component interventions in raising awareness about vaccines.^50^

### Access to vaccination

Unlike structured childhood vaccination programs, administration of adult vaccines in Canada is less routine and highly dependent on the actions of primary health care providers.^51^ An important barrier to vaccination is lack of access to vaccination (mentioned in 38% of studies). This barrier can be addressed by informing and referring people to health care professionals who have access to vaccines and are authorized to provide vaccination services. Most recommended vaccines are available at medical clinics, community pharmacies, community health centres, public health departments and travel clinics. Efforts are under way to evaluate an embedded community pharmacy-based approach aiming to improve vaccination rates in Canada utilizing pharmacist-delivered communicating and funding strategies.^52^ According to 2018 Canadian survey data, more Canadian adults received their influenza vaccination at a pharmacy than any other location.^53^ Recent reports highlight that pharmacists are well placed to improve access to vaccinations by using the annual influenza vaccination time to review and provide other adult immunizations as well.^54^

### Fear of adverse reactions

The second most common barrier identified was fear of adverse reactions. Despite overwhelming evidence supporting the safety of vaccines, lack of confidence in vaccine safety remains a major barrier to vaccination. Concerns range from fear of mild expected reactions such as injection site pain and redness to more serious but rare reactions such as anaphylaxis. Fears have been compounded by the spread of misinformation that vaccines can cause serious health problems such as developmental disorders or the very disease the vaccine is meant to protect against.

Seventy percent of Canadians use online resources for medical or health-related information.^55^ Thus, educating patients and providing reliable sources of information may limit the need for Canadians to seek out information from unreliable Internet sources, which can be misleading or inaccurate. Health care provider transparency about potential adverse effects can build trust and has been shown to lower perceived risk.^56^ Furthermore, it has been demonstrated that providing information about the adverse reaction reporting system may increase trust and vaccine acceptance among adults.^57^

Some individuals believe that the risk of contracting a vaccine-preventable disease is lower than the risks of experiencing a severe adverse reaction from a vaccine. A randomized controlled trial showed that stories and images highlighting the beneficial impact of vaccination on such diseases improved attitudes towards vaccination, especially among vaccine-hesitant individuals. Although encouraging, evidence is needed to confirm the effectiveness of storytelling in changing people’s intentions to vaccinate.^58^ According to a survey of primary care physicians in the United States, “the most common communication practices deemed very effective for convincing skeptical parents were personal statements by physicians about what they would do for their own children and about their personal experiences with vaccine safety among their patients.”^52^ Similar strategies can help counter antivaccine sentiments, which was another prominent barrier in this review (24%). Additionally, health care providers should leverage their position as trusted vaccine resources for adults by discussing expected adverse reactions, explaining adverse reaction management and correcting misconceptions. Canadians identify health care providers (HCPs) as their most trusted source for vaccine information, and research has shown that HCP recommendations are one of the strongest predictors of vaccine acceptance.^59,60^

### Financial barriers

Financial barriers are important concerns that affect immunization coverage in Canada. Vaccine recommendations in Canada are made by the National Advisory Committee on Immunization (NACI), while vaccine programs along with the decisions on vaccine coverage are implemented separately by each province or territory.^61^ Most vaccines are provided free for Canadians, as these vaccines have proven to be cost-effective for the health care system. However, evaluation of the economic impact takes time and results in some new vaccines not being covered for all individuals. A practical cost consideration is lack of access to local clinics with open extended hours in a geographic area. As a result, patients have to plan ahead and consider taking time off work to follow the recommended vaccination schedule.^62^ Most cost-associated barriers were in correlation with the HPV vaccination, as 1 dose of HPV costs about $185 and patients need 3 doses to gain immunity from 9 strains of HPV.^63^ Influenza vaccination, when it is not publicly covered, costs about $25 to $30/dose.^64^

### Awareness of vaccine existence

A survey conducted by Halperin et al.^39^ revealed that knowledge among adults about the Tdap vaccine was low, and only 36% of participants reported being aware that Tdap was recommended for all adults. Results of a cross-sectional study by Tatar et al.^35^ showed a positive correlation between HPV vaccine acceptance and knowledge about HPV and having discussed the HPV vaccine with their health care provider. Events such as the National Immunization Awareness Week (NIAW) help highlight the importance of immunization and the impact of vaccines on preventing illness and death. Multiple organizations in Canada and around the world participate in this annual event to raise awareness about vaccines and improve vaccination rates. The Canadian government has committed millions of dollars to immunization initiatives for surveillance, education, outreach and guidance on the use of vaccines.^65^ A study conducted by Shen and Dubey^6^ suggests patients are not discussing vaccines early enough with their health care providers. When pharmacists discuss vaccination status and provide information to patients, vaccination rates improve.^66^

### Resilience of older adults

Immunosenescence, weakened immune function due to natural aging, results in increased susceptibility to infectious diseases, especially in those with underlying chronic illnesses.^67^ As older adults are at higher risk of complications due to impaired immune function and comorbidities, it is essential to develop messaging and strategies to improve vaccine uptake in this population.^68^ The misconception of natural immunity to the vaccine-preventable disease due to age can be addressed by health care professionals providing education on nonvaccination risks, as recommended by the Canadian Public Health Agency.^66^

## Limitations

This review had several limitations. As most of the included studies were related to influenza and HPV vaccines, which are inactivated, it is possible the barriers may differ if a live vaccine were evaluated, or for other types of vaccines. Furthermore, the barriers we found are a compilation of general public and health care professionals (who would presumably be better informed). When quantifying the barriers to vaccination, we reported crude rates of studies (rather than individuals) reporting the various barriers. Finally, this review does not include literature describing barriers to relatively newly developed vaccines, such as the COVID-19 vaccine.

## Conclusion

Barriers to vaccination among Canadians and recent immigrants continue to challenge our health care system and contribute to vaccine hesitancy. Although decision-making regarding vaccination is complex and can be impacted by a number of factors, awareness of common barriers to vaccination has informed the development of strategies to improve vaccination uptake. Interventions to support vaccine-hesitant Canadians require effort and collaboration across all levels of our health care system. As trusted and accessible professionals, pharmacists are encouraged to incorporate vaccine assessments, preventive health and educational initiatives in their practice that may improve accessibility to vaccine services. ■

## Supplemental Material

sj-pdf-1-cph-10.1177_17151635221090212 – Supplemental material for Barriers to adult vaccination in Canada: A qualitative systematic review Click here for additional data file. Supplemental material, sj-pdf-1-cph-10.1177_17151635221090212 for Barriers to adult vaccination in Canada: A qualitative systematic review by Doris Stratoberdha, Barbara Gobis, Adrian Ziemczonek, Jamie Yuen, Annita Giang and Peter J. Zed in Canadian Pharmacists Journal / Revue des Pharmaciens du Canada
