# Supplementary material for: Barriers to adult vaccination in Canada: A qualitative systematic review
Source: Can Pharm J (Ott). 2022 Jun 6;155(4):206–18. doi: 10.1177/17151635221090212 (PMC9266373; doi:10.1177/17151635221090212)
Supplement: sj-pdf-1-cph-10.1177_17151635221090212 – Supplemental material for Barriers to adult vaccination in Canada: A qualitative systematic review [file sj-pdf-1-cph-10.1177_17151635221090212.pdf]

APPENDIX 1 Search terms and Boolean operators used in MEDLINE and EMBASE electronic databases

1. exp Vaccines, Inactivated/ or exp Vaccines, Subunit/ or exp Rabies Vaccines/ or exp Escherichia coli Vaccines/ or exp Typhoid-Paratyphoid Vaccines/ or exp Cancer Vaccines/ or exp Pneumococcal Vaccines/ or exp Bacterial Vaccines/ or exp Poliovirus Vaccines/ or exp Vaccines, Conjugate/ or exp Papillomavirus Vaccines/ or exp Marek Disease Vaccines/ or exp Viral Hepatitis Vaccines/ or exp Vaccines/ or exp Tuberculosis Vaccines/ or exp Vaccines, Attenuated/ or exp Fungal Vaccines/ or exp Alzheimer Vaccines/ or vaccine.mp. or exp Malaria Vaccines/ or exp Pseudorabies Vaccines/ or exp Vaccines, Marker/ or exp Shigella Vaccines/ or exp Staphylococcal Vaccines/ or exp Hepatitis B Vaccines/ or exp Vaccines, DNA/ or exp Vaccines, Virus-Like Particle/ or exp Influenza Vaccines/ or exp Vaccines, Edible/ or exp SAIDS Vaccines/ or exp Herpesvirus Vaccines/ or exp Rotavirus Vaccines/ or exp AIDS Vaccines/ or exp Respiratory Syncytial Virus Vaccines/ or exp Lyme Disease Vaccines/ or exp Salmonella Vaccines/ or exp Vaccines, Combined/ or exp Ebola Vaccines/ or exp Streptococcal Vaccines/ or exp Cytomegalovirus Vaccines/ or exp Cholera Vaccines/ or exp Viral Vaccines/ or exp Hepatitis A Vaccines/ or exp Vaccines, Acellular/ or exp Japanese Encephalitis Vaccines/ or exp Herpes Simplex Virus Vaccines/ or exp Pseudomonas Vaccines/ or exp Meningococcal Vaccines/ or exp Rickettsial Vaccines/ or exp Haemophilus Vaccines/ or exp Anthrax Vaccines/ or exp Vaccines, Contraceptive/ or exp Dengue Vaccines/ or exp Vaccines, Virosome/ or exp Vaccines, Synthetic/ or exp West Nile Virus Vaccines/ or exp Adenovirus Vaccines/ or exp Parainfluenza Vaccines/ or exp Protozoan Vaccines/ or exp Leishmaniasis Vaccines/ or exp Diphtheria-Tetanus-acellular Pertussis Vaccines/ or exp Vaccines, Live, Unattenuated/
2. exp Mass Vaccination/ or exp Vaccination Refusal/ or exp Anti-Vaccination Movement/ or exp Vaccination Coverage/ or exp Vaccination/ or vaccination.mp.
3. vaccinations.mp. or exp Vaccination/
4. exp Vaccination/ or vax.mp. or exp Viral Vaccines/
5. barrier.mp.
6. barriers.mp.
7. canada.mp. or exp Canada/
8. canadian.mp.
9. exp Immunization/ or immunize.mp.
10. exp Immunization, Passive/ or exp Immunization, Secondary/ or exp Immunization/ or exp Immunization Schedule/ or exp Immunization Programs/ or immunization.mp.
11. 1 or 2 or 3 or 4 or 9 or 10
12. 5 or 6
13. 7 or 8
14. 11 and 12 and 13
15. Remove duplicates from 14

APPENDIX 2 Literature search and flow diagram of included studies

Identification

Records identified through database searching (n = 428)

Additional records identified through other sources (n = 0)

Records after duplicates removed (n = 306)

Screening

Records screened (n = 306)

Records excluded (n =259)

Eligibility

Full-text articles assessed for eligibility (n = 47)

Full-text articles excluded, with reasons (n = 13)

Included

Studies included in literature review (n = 34)

Stratoberdha D, et al. Barriers to adult vaccination in Canada: a qualitative systematic review. Can Pharm J (Ott) 2022;155. DOI: 10.1177/17151635221090212.
